# Plant stem tissue modeling and parameter identification using metaheuristic optimization algorithms

**DOI:** 10.1038/s41598-022-06737-z

**Published:** 2022-03-10

**Authors:** Mohamed S. Ghoneim, Samar I. Gadallah, Lobna A. Said, Ahmed M. Eltawil, Ahmed G. Radwan, Ahmed H. Madian

**Affiliations:** 1 Nanoelectronics Integrated Systems Center (NISC), Nile University, Giza, Egypt; 2 Electrical Engineering and Computer Science Department, University of California-Irvine, Irvine, USA; 3 King Abdullah Univ. of Science and Technology, Thuwal, Saudi Arabia; 4 Engineering Mathematics and Physics Department, Cairo University, Giza, Egypt; 5 School of Engineering and Applied Sciences, Nile University, Giza, Egypt; 6 Radiation Engineering Department, Egyptian Atomic Energy Authority NCRRT, Cairo, Egypt

**Keywords:** Electrical and electronic engineering, Biological models

## Abstract

Bio-impedance non-invasive measurement techniques usage is rapidly increasing in the agriculture industry. These measured impedance variations reflect tacit biochemical and biophysical changes of living and non-living tissues. Bio-impedance circuit modeling is an effective solution used in biology and medicine to fit the measured impedance. This paper proposes two new fractional-order bio-impedance plant stem models. These new models are compared with three commonly used bio-impedance fractional-order circuit models in plant modeling (Cole, Double Cole, and Fractional-order Double-shell). The two proposed models represent the characterization of the biological cellular morphology of the plant stem. Experiments are conducted on two samples of three different medical plant species from the family *Lamiaceae*, and each sample is measured at two inter-electrode spacing distances. Bio-impedance measurements are done using an electrochemical station (SP150) in the range of 100 Hz to 100 kHz. All employed models are compared by fitting the measured data to verify the efficiency of the proposed models in modeling the plant stem tissue. The proposed models give the best results in all inter-electrode spacing distances. Four different metaheuristic optimization algorithms are used in the fitting process to extract all models parameter and find the best optimization algorithm in the bio-impedance problems.

## Introduction

Natural products derived from plants, animals, and minerals have been the primary method for treating human diseases. Throughout history, medical plants have become in high demand for their efficiency in treating and preventing diseases^1^. Important compounds could be derived from their leaves, stems, roots, fruits or used as a whole plant. Nowadays, medical plant extracts became essential in most chemical medicines and commercial products^2^. The global market for botanical and plant-derived drugs, according to a study by BCC Research (Business Communications Company as a leading market information resource), will grow from 29.4 billion$ in 2017 to around 39.6 billion$ by 2022 with a compound annual growth rate (CAGR) of 6.1% for the period of 2017–2022^3^, where medical plants represent 10% of Vascular plants with around of 350,000 species.

According to the Angiosperm Phylogeny Group classification (APG) in 2016, 25 of 416 families of flowering plants are crucial for human needs in medicine^4^, where they hold specific herbal properties such as anti-oxidant, anti-bacterial and anti-nociceptive effects. One of the most prominent families belongs to the flowering plants is *Lamiaceae*. It is very distinctive and known for its useful constituents in pharmacological and therapeutic treatments and its contribution to different biological activities^5^. It consists of around 7000 species with 250 genera that are diverse and spreads widely in different ecosystems.

Species that belong to the Lamiaceae family contain secondary metabolites with antimicrobial, antiviral, anticancer, anti-inflammatory and antioxidant properties where it is mainly studied as a natural antioxidant source. They are well known for their biochemical extracts and essential oil that is found in leaves, stem, and flower^5^. Most aromatic existent family species have proved efficient results in treating gastrointestinal tract disorders and diseases that affect the cardiovascular system and upper respiratory tract, such as “Arterial HyperTension(AHT)”^6^. The critical compounds derived from this family include tannins, polyphenols, flavonoids, alkaloids, and terpenoids, which contributes significantly to the importance of the Lamiaceae family in Health treatment^5^. The flavonoids have proved to be associated with lower coronary heart disease mortality^7^. Terpenes are responsible for antitumor, antibacterial, cardiotonic and anti-inflammatory effects. Tannin helps prevent or treat atherosclerosis, and alkaloids are beneficial in treating cardiovascular and central nervous systems diseases^8^.

*Origanum majorana*, *Salvia officinalis* L. and *Lavandula* are common species included in the *Lamiaceae* family. They are medical aromatic herbs that grow and are commonly used in Egypt and well known in North Africa, the Mediterranean and Western Asia^9–11^. Besides being used in commercial products and food industry, they proved to be beneficial in traditional medicine due to their antimicrobial, antiviral, anti-inflammatory, antifungal and antioxidant properties^12^.

*Origanum majorana*, known as Marjoram, is used as a whole plant, ground or even as a source for essential oils. The addition of Marjoram to vegetables during storage provides protection against Oxidation, Light damage, pigment degradation and spoilage fungus^9^. Marjoram has proved efficient healing role of some diseases, such as treating obesity due to hyperlipidaemia^13^. Ethanol extraction from Marjoram’s stem is used in the prevention of cancer and carcinogenesis mutations. Its oil may improve asthmatic patients’ health condition and is used in enhancing liver and kidney activities^14^. In general, it is deployed as a safe traditional medicine that is used in curing coughs, indigestion, rheumatism, toothache and heart conditions^9^.

Salvia is the largest genus in the Lamiaceae family known as Common Sage (*Salvia officinalis* L.)^5^. Extracts of this plant were found to reduce the growth of different kinds of cancers and reduce mutations^12^. Its antioxidant properties have a vital role in reducing the development of cardiovascular and neurological diseases. Some of its extracts have anti-inflammatory, antibacterial and anti-malarial effects that could be deployed in clinical drugs to dispose of the undesired side effects^10^.

*Lavandula* is a common genus in the Lamiaceae, known for its strong fragrance and the usage of lavender oil for various health conditions such as stress, fatigue, and it is also common in aromatherapy^15^. It was deployed in medicine for kidney and stomach issues, and provided satisfying results for the central nervous system. Also, from its benefits in the medical field are mean blood pressure reduction, Pulmonary (related to Lung) sickness therapy, and Neuro-psychiatric^11^.

The plants’ conditions under different environments are monitored through non-destructive and destructive measurements. Monitoring plants helps in reducing damage, preventing diseases and increasing plant yield. Bio-impedance is the produced electrical impedance from the excitation of the biological cell through an AC signal (voltage or current) stimulus^16^. Tissue impedance changes depend on extra- and intra-cellular resistance, health status, structure, morphology, type, location, chemical composition, and shape^17^. When the tissue is excited with an AC voltage signal, at low frequencies, the current paths through the extracellular fluid around the cells, while at higher frequencies, the current flows everywhere (through the cells), and that leads to lowering the impedance due to the capacitive nature of the cells^17,18^. According to these different responses, it is vital to use a wide range of frequencies to measure bio-impedance.

Bio-impedance measurements are used in the diagnosis of plants behaviour to certain conditions such as fruit maturity^19,20^, fruit ripening^21,22^, analyzing the effect of heating and freezing conditions on fruits^23^, measuring of root growth^24^, and determining the water content and characteristic analysis of the root zone^25^. Also, it is used to provide information about environmental change effect on fruits^26^. In^27^, the tissue damage of a bruised apple sample was determined by using electrical impedance. There are other contributions in using bio-impedance measurements for different applications such as blood glucose measurement^28^, monitoring insulin availability for personalized diabetes therapy^29^, Characterising red blood cell micro-circulatory parameters^30^, and tactile sensing bio-hybrid soft E-skin in soft robotics^31^. The heating and freezing of the plant tissue and their effect on the bio-impedance models’ parameters are discussed in^23^, where the impedance drops as the temperature increases. Impedance increased in the samples that suffered from freezing conditions, indicating cellular damage due to ice generation.

Different bio-impedance circuit models were proposed to represent the electrical characteristics of the biological cell of plants^17^. In 1940, it was the first representation for the biological tissue by introducing the single dispersion Cole-impedance model^32^. It became the most popular and commonly used models due to its simplicity and fitting accuracy^33^. In 1969, the Hayden model was introduced to provide representation for cell components^34^, but showed some faults in fitting due to the missing of vacuole representation^35^. In 1990, the double-shell model was introduced to overcome the defects of the Hayden model by adding the representation of vacuole in the proposed model^16,18^. However, the double-shell model showed many defects in fitting at low frequencies^35^. Then the second generation of single dispersion Cole-impedance model, which is the double dispersion Cole-impedance model, was presented to improve the representation accuracy of impedance over broadband frequencies^36^. In^35^, the Hayden, simplified Hayden and double-shell models were reintroduced into the fractional-order form to add more flexibility in the fitting process and overcome the integer defects. Hayden model was used in the characterization of various plants such as carrot roots and cabbage leaves in^16^. Plant shoots and Stem were modeled for the Soybean plant using general models that do not clearly describe the stem functions^37^. To the author’s knowledge, there was no previous attempt to model or characterize medical plants in general, including the *Lamiaceae* family.

Fractional calculus (FC) is the study that governs the operation of integrals and derivatives of non-integer order, where traditional calculus is a small subset of it. Fractional order modeling most notable benefits are the memory dependency in the fractional derivative definition, and adding more degree of freedom that increases the controllability and flexibility of the system through the extra parameter from the derivative order^38,39^. Recently, fractional calculus become the pioneer in many fields such as control systems^40^, filters^39,41^, robotics^42^, encryption^43^, chaotic systems^44^, bio-engineering^45,46^, and super-capacitor modeling^47^.

Recently, metaheuristic optimization algorithms, which are inspired by natural phenomena, showed a successful employment for the bio-impedance parameter extraction problems^48,49^. Metaheuristics are used to mimic the intelligence-gathering behavior of water cycle^50^, pollination process in the plant^49^, chicken behavior in the swarm^51^, red fox searching for food, hunting and escaping from hunters^52^, black widow spider mirage^53^, Harris Hawks during chasing of the prey^54^, elephants herd and the distance between them^55^, hunting process of the grey wolf^56^, and many others. They are used to overcome the defecates and difficulties that face the traditional optimization methods^49^. In^33^, flower pollination algorithm (FPA) and moth flame optimization (MFO) were used to extract the Cole-impedance model parameters and compared with the traditional nonlinear least square (NLS). FPA and MFO showed their superiority over the NLS method in fitting the measured data and accuracy of the extracted parameters. Also, the FPA achieved the best accuracy and consistency over the other employed optimization (NLS,MFO), while the NLS technique was the fastest. In^49^, six different metaheuristic optimizations were used to extract the Cole-impedance model parameters using two different datasets magnitude only impedance measurements and complex impedance measurements. It was found that Cuckoo search optimization (CS) and FPA algorithms had a better fitting for the experimental datasets, less error and higher consistency than the other used algorithms. FPA, CS, and MFO algorithms were used in^45^ to extract the Cole-impedance model parameters using an alternative way to measure the bio-impedance (differentiator circuit). It was concluded that CS and FPA algorithms had a quite similar performance, where CS converges faster, and FPA takes less run time. FPA showed a reasonable parameter extraction over CS and MFO.

Optimization algorithms are recognized as a soft computing method used to solve complex problems. Soft computing is concerned with approximate models and controlling complex systems, as it is tolerant to imprecision, uncertainty and approximations. Soft computing is a combination of optimization algorithms, in addition to artificial neural networks and machine learning algorithms that are used for decision-making^57^, identification^58^, and predictions support^59^.

In this paper, two new Fractional-order electrical impedance models are proposed for plant stem representation. The stem impedance is measured using SP150 for two samples of three medical plants (Marjoram, *Salvia officinalis* L., *Lavandula*) from *Lamiaceae* plant family. The measured impedance data are fitted on three commonly used bio-impedance models with plants (Cole, double Cole and Fractional-Order double-shell), and compared with the two proposed models. Then the models’ parameters are extracted using four metaheuristic optimization algorithms [FPA, CS, WCA and Chicken swarm optimization (CSO)]. The Nyquist plot is plotted for the measured and the fitted data for all models. The error between the measured and fitted data is calculated to find the best model and the best optimization algorithm.

This paper is organized as follows: Section “Stem modeling” briefly describes the plant stem anatomy and the role of each stem layer, it also shows the impedance model circuit’s analysis and their representation. Section “Problem definition” illustrates the problem formulation. Section “Experimental results and discussion” provides the experimental results and discussion. Finally, Section “Conclusion” concludes the paper.

## Stem modeling

### Stem tissue structure

**Figure 1:**
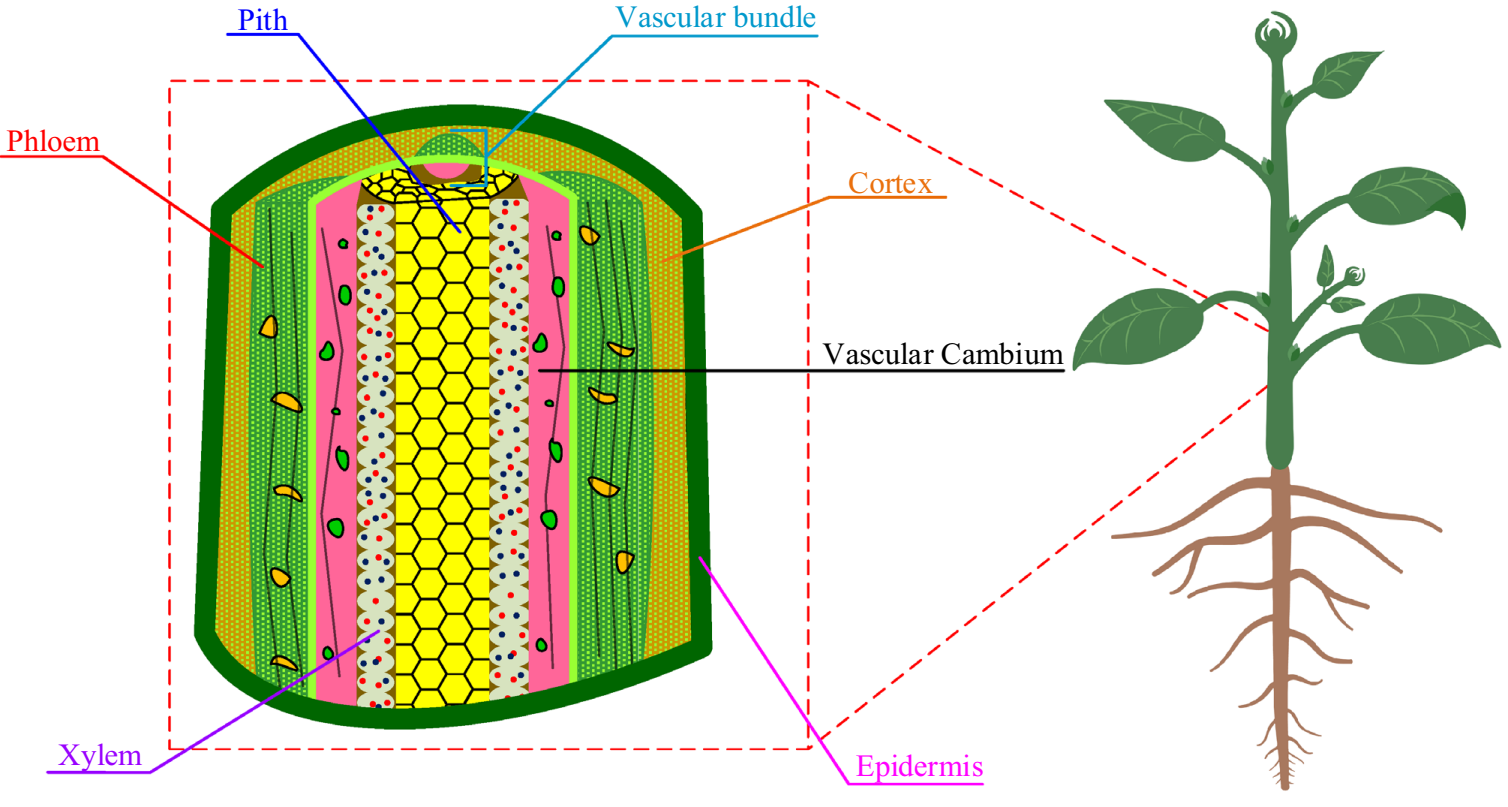
The vertical cross-section of a typical plant stem structure consists of a core, defined as the pith surrounded by a group of vascular bundles enclosed with the cortex. Those are encapsulated with the epidermis.

Plant Stem plays a vital role in the growth and protection of the plant, providing support to the plant weight. It bears the flowers and leaves of the plant and acts as a transportation channel for water, nutrients and food through all the plant parts. The green stems participate in the Plant’s Photosynthesis process^60^. Monitoring the plant stem helps to investigate the plant’s condition, such as transpiration rate (water flow) and nutrient concentration. It could also act as an indication to the soil state^37^. For medical plants, some beneficial compounds are extracted from the plant stem, such as ethanol that has antioxidant and anti-gout activity; it also contributes to the production of some essential oils^61^. The stem (see Fig. 1) includes multiple layers that depend on the structure of the plant and its growing conditions. It mainly consists of Vascular, ground and Epidermis systems^60^. The Vascular system is composed of Xylem and Phloem as Complex cells. The Xylem is responsible for transferring water and nutrients from the roots along the whole stem and into the leaves. It is a one-directional tube that consists of smaller tubes connected through a gate. The Phloem is a bidirectional transportation system that transports food and organic materials from the green parts to the rest of the plant. The Phloem and Xylem are grouped in vertical strands called vascular bundles and are separated by a layer of cells named cambium^37,60^.

**Figure 2:**
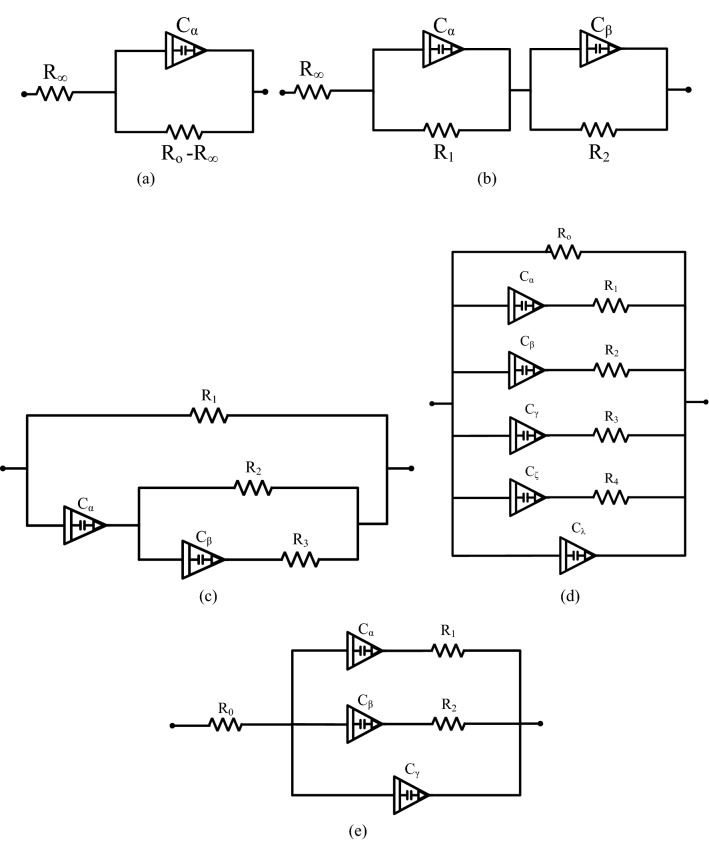
The electrical circuit of bio-impedance models (**a**) Single dispersion Cole-impedance model (**b**) Double dispersion Cole-impedance model (**c**) Fractional-order Double Shell model (**d**) Proposed Stem model: represents the cortex, vascular cambium, phloem, and xylem with a series resistor and fractional-order capacitor. While the pith is represented with a fractional-order capacitor and the epidermis with a resistor. (**e**) The proposed simplified stem model: grouped the vascular bundle elements (vascular cambium, phloem, and xylem) into a single resistor and fractional-order capacitor.

**Figure 3:**
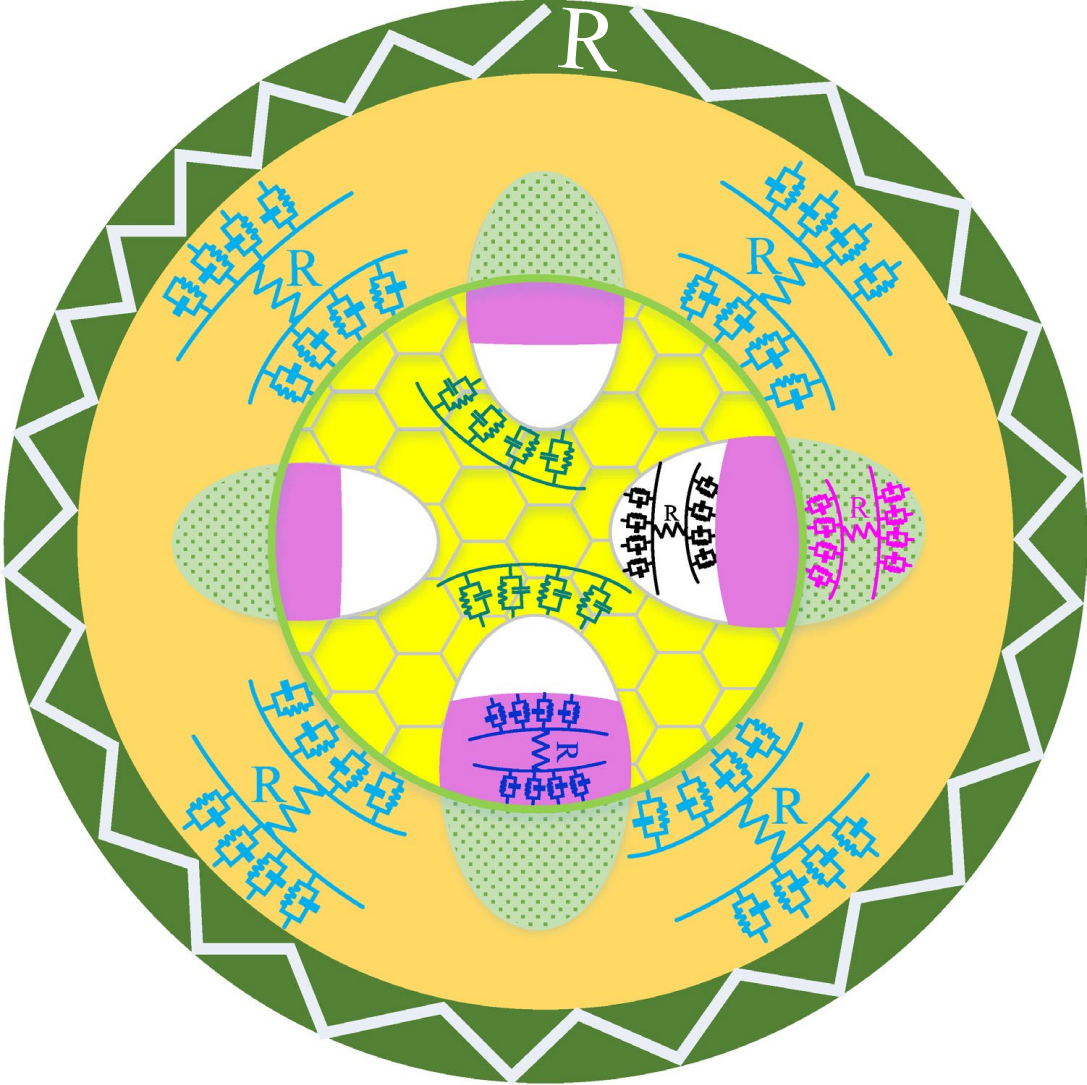
Plant stem electrical equivalent circuit. The epidermis is a single protective hard layer represented with a resistor. While, the cortex and vascular bundle elements consist of a membrane and inner fluid responsible for storing carbohydrates and the flow of water, nutrients, food, and organic materials represented by a resistor and a fractional-order capacitor. The pith is a spongy structured tissue described by a fractional-order capacitor.

The epidermis in Fig. 1 is the outer layer that covers the stem with a rigid structure and waxy appearance in some plant species. It protects the stem against injury, infection and water loss^60^. It also acts as a controller of the gas, water and nutrients exchange with the surrounding environment. The epidermis evolved various features, such as some specific cell types and guard cells, to adjust to its various functions. The cell’s shapes and functions are developed according to their growing circumstances^62^.

The Plant ground parts are responsible for the stem support, where it consists of the Pith and the cortex that is located between the vascular bundle and the epidermis. The tissue cells of the cortex may include essential oils, tannins and stored carbohydrates. The Pith is at the centre of the stem with a soft spongy structure. It contributes to the storage of nutrients and minerals. For some plants, the stem could harden and then decomposes to produce a hollow shaped stem^60^.

### Electrical modeling

#### General bio-impedance models

The biological cell cannot be dealt with as a homogeneous medium, consisting of various complex elements^35^. When current migrates through a cell, it is attenuated by existent water electrolytes, and intracellular and extracellular components. The Cole-impedance model shown in Fig. 2a proved to produce a good fit to experimental impedance data when applied to different tissues. It was initially proposed as a general model. Then it was applied more specifically to describe plant status. The Cole-impedance model is represented as follows:

1 $$\begin{aligned} Z(s)=R_{\infty }+\frac{R_{o}-R_{\infty }}{1+S^{\alpha } C_{\alpha } (R_{o}-R_{\infty })}, \end{aligned}$$

where $R_o$ represents the resistance at low frequency, $R_\infty$ represents the high-frequency resistance and $\alpha$ represents the Constant phase element(CPE) order. The Cole-impedance model is used to fit the measured data of various types of tissues such as shoots and leaves tissues in^63^.

It was also used to study the effect of freezing–thawing on eggplant, maturity measurements of fruits and vegetables^64^. In^65^, the ripening of fruits was investigated using various models. In^17^, the Cole-impedance model was used to study the photosynthetic activity in plants during illumination and darkness. Although this model gave good results for most of the experimented tissues, it did not provide an explanation for the operating mechanism in the cell.

To provide a better representation for cell components in complex materials, the Cole-impedance model was expanded as shown in Fig. 2b into double Dispersion Cole impedance model where its impedance representation is as follows:

2 $$\begin{aligned} Z(s)=R_{\infty }+\frac{R_{1}}{1+S^{\alpha } R_{1} C_{\alpha }}+\frac{R_{2}}{1+S^{\beta } R_{2} C_{\beta }}. \end{aligned}$$

The double dispersion Cole impedance model was used as a representation of the plant stem in^37^. It was used to fit measurement data of various fruits and vegetables such as banana, cucumber and oranges in^35^, where different models were compared, and the double Cole impedance model provided the best results. The double Cole impedance model could be used as an indicator of frost hardening in shots of Scots pine^64^.

To accurately describe the plant tissue cell’s components, the Double-shell model was developed to provide a representation of the cell Vacuole. The fractional-order Double-shell model shown in Fig. 2c was firstly introduced in^35^ and its impedance is described as follows:

3 $$\begin{aligned} \begin{aligned} Z(s)&= \frac{R_{1}\left( S^{\alpha +\beta } C_{\beta } C_{\alpha } R_{2} R_{3}+S^{\alpha } C_{\alpha } R_{2}+S^{\beta } C_{\beta }\left( R_{2}+R_{3}\right) +1\right) }{S^{\alpha +\beta } C_{\beta } C_{\alpha }\left( R_{2} R_{3}+\left( R_{2}+R_{3}\right) R_{1}\right) +S^{\beta } C_{\beta }\left( R_{2}+R_{3}\right) +S^{\alpha } C_{\alpha }\left( R_{1}+R_{2}\right) +1}, \end{aligned} \end{aligned}$$

where $R_1$ represents the extracellular resistance, $R_2$ represents the intracellular resistance, $R_3$ and $C_{\beta }$ represent the Vacuole resistance and capacitance respectively, and $C_{\alpha }$ represents the plasma membrane capacitance. The double-shell model is used in studying different plant condition such as ripening, heating and Freezing, but proved to be most efficient in plants ripening^17^. It was also used as a representation for the plant stem structure^37^.

#### Proposed bio-impedance models

The proposed electrical impedance model in Fig. 2d characterizes the plant stem. The Epidermis (see Fig. 3) is a hard protective layer; in most cases, it consists of a single layer of cells represented by an electrical resistor $R_o$. The Xylem, Phloem, and the Cambium (Bundle) are each represented by a resistor and capacitor in series as they have a tube-like structure. Also, the Cortex has the exact representation. While the Spongy pith is represented by a capacitor $C_\lambda$. The electrical impedance of the stem model is described as following:

4a $$\begin{aligned} Z(S)&=\frac{R_{o}(1+S^{\alpha } R_{1} C_{\alpha })(1+S^{\beta } R_{2} C_{\beta })(1+S^{\gamma } R_{3} C_{\gamma })(1+S^{\zeta } R_{4} C_{\zeta })}{Z_f+Z_k+Z_p+Z_m}, \end{aligned}$$

4b $$\begin{aligned} Z_f(S)&=(S^{\alpha } R_{1} C_{\alpha }+S^{\alpha } R_{o} C_{\alpha }+1)(S^{\beta } R_{2} C_{\beta }+1)(S^{\gamma } R_{3} C_{\gamma }+1)(S^{\zeta } R_{4} C_{\zeta }+1), \end{aligned}$$

4c $$\begin{aligned} Z_k(S)&=R_{o}(1+S^{\alpha } R_{1} C_{\alpha })(S^{\gamma +\beta } R_{3} C_{\gamma } C_{\beta }+S^{\gamma +\beta } R_{2} C_{\beta } C_{\gamma }+S^{\beta } C_{\beta }+S^{\gamma } C_{\gamma })(S^{\zeta } R_{4} C_{\zeta }+1), \end{aligned}$$

4d $$\begin{aligned} Z_p(S)&=(1+S^{\beta } R_{2} C_{\beta })(1+S^{\gamma } R_{3} C_{\gamma })(1+S^{\alpha } R_{1} C_{\alpha }) S^{\zeta } C_{\zeta } R_{o}, \end{aligned}$$

4e $$\begin{aligned} Z_m(S)&=S^{\lambda } C_{\lambda } R_{o}(1+S^{\alpha } R_{1} C_{\alpha })(1+S^{\beta } R_{2} C_{\beta })(1+S^{\gamma } R_{3} C_{\gamma })(1+S^{\zeta } R_{4} C_{\zeta }), \end{aligned}$$

The proposed model relates the biological changes that affect the plant stem during testing to the bio-impedance data.

The proposed stem model is simplified as in Fig. 2e by representing the vascular bundle by a single branch of series resistor and capacitor. Its electrical impedance is described as follows:

5a $$\begin{aligned} Z(s)&=R_o+\frac{(S^\beta C_\beta R_2 +1) (S^\alpha C_\alpha R_1 +1)}{Z_k+Z_m}, \end{aligned}$$

5b $$\begin{aligned} Z_k(S)&=S^{\alpha +\beta } C_\alpha C_\beta (R_1+R_2)+S^\beta C_\beta +S^\alpha C_\alpha , \end{aligned}$$

5c $$\begin{aligned} Z_m(S)&=S^\gamma C_\gamma (S^{\alpha +\beta } C_\alpha C_\beta R_1 R_2 +S^\beta C_\beta R_2 +S^\alpha C_\alpha R_1 +1), \end{aligned}$$

where $R_o$ represents the Epidermis,$R_1$ and $C_\alpha$ represents the Cortex resistance and capacitance respectively, $R_2$ represents the resistance, and $C_\beta$ represents the capacitance of the vascular bundle and $C_\gamma$ represents the Pith capacitance.

## Problem definition

**Figure 4:**
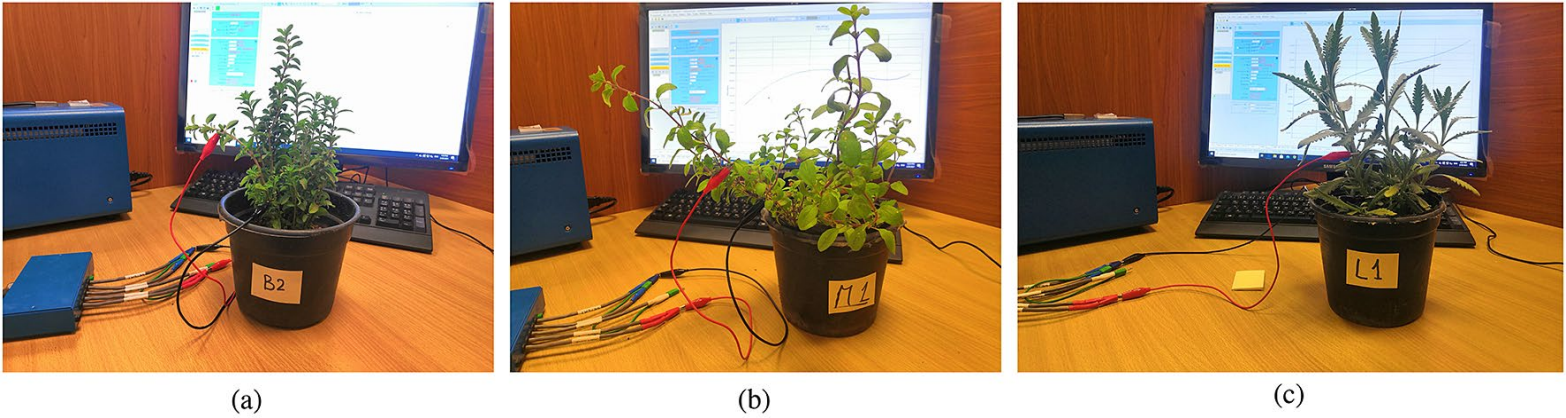
Experimental samples setup for (**a**) *Origanum majorana*, (**b**) *Salvia officinalis* L. and (**c**) *Lavandula*. Two electrodes are placed to the employed samples’ stem with a distance 5 cm and 10 cm and then the impedance is tested by standard Impedance Spectroscopy (SP-150).

Due to the distinguished medical benefits of the *Lamiaceae* family plants and their availability, three species with common traits are selected in this study. The selected plants were purchased from the market and cultivated by an outsource company (Safwa For Agriculture) and supervised by the support services office at Nile University that complies with institutional, national, and international guidelines and legislation. The three plants are identified as *Origanum majorana*, *Salvia officinalis* L., and *Lavandula*. Two sample plants of each species are employed in the experiment. Electrochemical workstation, commonly used in impedance analyzing, (SP150) is used to measure the impedance of the plants at room temperature of 25 °C for a frequency range from 10 Hz to 100 kHz. Electrodes are placed along the plant stem with a distance of 5 cm and 10 cm apart from each other noninvasively for each sample, as shown in Fig. 4, to verify the effect of electrodes separation on the observed results. The two experiments are consecutively done on each sample to prevent any changes in the impedance. The applied sinusoidal voltage excitation is $V_{rms}=20mV$ with no DC offset. The number of the measured points is 80 points per decade. Then the log is imported to MATLAB to run post-processing.

Post-processing is conducted on the measured impedance data using four metaheuristic optimization algorithms to extract the parameters of the employed models. The applied algorithms are adopted according to the literature, where they proved to output satisfying results. It is essential to precisely define the factors influencing the optimization algorithm’s result to obtain optimal bio-impedance models’ parameters. The factors include the objective function, number of search agents, runs and iteration, the upper and lower boundaries, and the vector of optimized variables.

1. The objective function represented in Eq. (6) is the sum of the absolute error between the estimated impedance from the model and the measured impedance of the sample for each frequency point.
  6 $$\begin{aligned} min | \frac{Z_{model}(x) - Z_{measured}}{Z_{measured}}| = min \sum ^{n}_{i} |\frac{Z_{model}(x_i) - Z_{measured}(i)}{Z_{measured}(i)}|, \end{aligned}$$
  where *x* is the impedance parameters of each model depending on the problem size, $Z_{model}(x)$ is the impedance equation of the models, while $Z_{measured}$ is the measured response of the sample. *n* is the total number of the measured points.
2. The number of search agents used in the optimization is 60 and runs for 100 independent runs through 1800 iterations for all the tested samples.
3. The search agents search for the best solution in a region defined between a lower (LB) and an upper (UB) boundary defined differently for each model are shown in Table 1.
4.
  - For Cole-impedance model, the impedance parameters are [$\alpha$, $R_{\infty }$, $R_o$, *C*_*α*_].
  - the Double dispersion Cole impedance model parameters are [$\alpha$, $\beta$, $R_\infty$, $R_1$, $R_2$, $C_\alpha$, $C_\beta$].
  - The impedance parameters for Fractional-Order Double-shell model are [$\alpha$, $\beta$, $R_1$, $R_2$, $R_3$, $C_\alpha$, $C_\beta$]
  - The proposed Stem model parameters are [$\alpha$, $\beta$,$\gamma$,$\zeta$,$\lambda$, $R_o$, $R_1$, $R_2$,$R_3$, $R_4$, $C_\alpha$, $C_\beta$,$C_\gamma$,$C_\zeta$,$C_\lambda$].
  - The impedance parameters for the proposed simplified Stem model are [$\alpha$, $\beta$,$\gamma$, $R_o$, $R_1$, $R_2$, $C_\alpha$, $C_\beta$,$C_\gamma$].

**Table 1:** The lower boundary (LB) and the upper boundary (UB)for each model.

Models	Cole		Double Cole		Double-shell		Proposed stem model		Proposed simplified stem model	
	LB	UB	LB	UB	LB	UB	LB	UB	LB	UB
**Parameters**										
$\alpha$	0	1	0	1	0	1	0	1	0	1
$\beta$	–	–	0	1	0	1	0	1	0	1
$\gamma$	–	–	–	–	–	–	0	1	0	1
$\zeta$	–	–	–	–	–	–	0	1	–	–
$\lambda$	–	–	–	–	–	–	0	1	–	–
$R_\infty$	0	100 K$\Omega$	0	1 M$\Omega$	–	–	–	–	–	–
$R_o$	0	80 M$\Omega$	–	–	–	–	0	1 G$\Omega$	0	100 M$\Omega$
$R_1$	–	–	0	1 M$\Omega$	0	10 M$\Omega$	0	1 G$\Omega$	0	100 M$\Omega$
$R_2$	–	–	0	1 M$\Omega$	0	10 M$\Omega$	0	1 G$\Omega$	0	100 M$\Omega$
$R_3$	–	–	–	–	0	10 M$\Omega$	0	1 G$\Omega$	–	–
$R_4$	–	–	–	–	–	–	0	1 G$\Omega$	–	–
$C_\alpha$	0	$3 \mu \hbox {F}$	0	$4 \mu \hbox {F}$	0	$3\mu \hbox {F}$	0	$100\mu \hbox {F}$	0	$10\mu \hbox {F}$
$C_\beta$	–	–	0	$4 \mu \hbox {F}$	0	$3 \mu \hbox {F}$	0	$100\mu \hbox {F}$	0	$10\mu \hbox {F}$
$C_\gamma$	–	–	–	–	–	–	0	$100\mu \hbox {F}$	0	$10\mu \hbox {F}$
$C_\zeta$	–	–	–	–	–	–	0	$100\mu \hbox {F}$	–	–
$C_\lambda$	–	–	–	–	–	–	0	$100\mu \hbox {F}$	–	–

**Figure 5:**
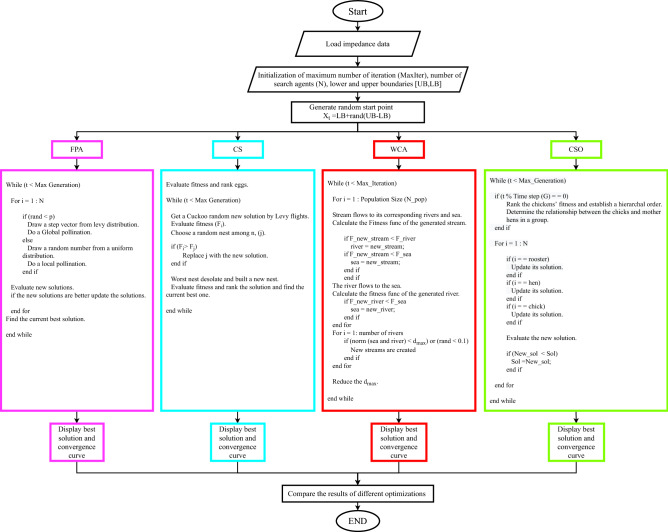
Summery flowchart for the different employed optimization process.

## Experimental results and discussion

In this section, The two proposed stem models are validated by fitting the measured data and using FPA, CS, CSO, and WCA optimization techniques in the models’ parameters extraction process. As mentioned in the literature, FPA and CS optimization algorithms are used before in bio-impedance parameter extraction problems and showed good performance, while WCA and CSO are used for the first time for such problems. The four algorithms are compared to select the most fitted algorithm in such a problem by studying the error and convergence curves. Figure 5 shows a flowchart that summarises the employed optimizations; more details about these optimizations can be found in^49–51^.

The measured data at a distance = 5 cm and 10 cm for the studied plant samples were fitted on three models (Cole, Double Cole, and Fractional-Order Double shell models) and the two proposed models (Stem and simplified stem models). The extracted models’ parameters by WCA are shown in Table 2.

**Table 2:** The extracted parameters from the measured plant stem samples using different optimization techniques.

Parameters	$\alpha$	$\beta$	$\gamma$	$\zeta$	$\lambda$	$R_\infty$	$R_o$	$R_1$	$R_2$	$R_3$	$R_4$	$C_\alpha$	$C_\beta$	$C_\gamma$	$C_\zeta$	$C_\lambda$
Models																
Cole																
Marjoram “5 cm”	0.6647	–	–	–	–	5.6834 (K$\Omega$)	202.28 (K$\Omega$)	–	–	–	–	4.2952 (nF)	–	–	–	–
Marjoram “10 cm”	0.73	–	–	–	–	4.9567 (K$\Omega$)	558.89 (K$\Omega$)	–	–	–	–	1.1493 (nF)	–	–	–	–
Salvia “5 cm”	0.7035	–	–	–	–	23.987 (K$\Omega$)	441.99 (K$\Omega$)	–	–	–	–	2.7149 (nF)	–	–	–	–
Salvia “10 cm”	0.7159	–	–	–	–	25.565 (K$\Omega$)	991.37 (K$\Omega$)	–	–	–	–	1.4455 (nF)	–	–	–	–
*Lavandula* “5 cm”	0.5016	–	–	–	–	9.4741 (K$\Omega$)	384.92 (K$\Omega$)	–	–	–	–	65.825 (nF)	–	–	–	–
*Lavandula* “10 cm”	0.5562	–	–	–	–	2.1391 (K$\Omega$)	604.51 (K$\Omega$)	–	–	–	–	12.135 (nF)	–	–	–	–
Double Cole																
Marjoram “5 cm”	1	0.6828	-	-	-	8.6531 (K$\Omega$)	-	31.628 (K$\Omega$)	194.2 (K$\Omega$)	-	-	67.17 (nF)	3.6101 (nF)	-	-	-
Marjoram “10 cm”	1	0.7156	–	–	–	0	–	38.239 (K$\Omega$)	523 (K$\Omega$)	–	–	1.6952 (nF)	1.3449 (nF)	–	–	–
Salvia “5 cm”	0.5117	0.7908	–	–	–	0	–	143.71 (K$\Omega$)	316.41 (K$\Omega$)	–	–	27.943 (nF)	1.819 (nF)	–	–	–
Salvia “10 cm”	0.8128	0.5932	–	–	–	0	–	757.92 (K$\Omega$)	213.18 (K$\Omega$)	–	–	0.8319 (nF)	8.0292 (nF)	–	–	–
*Lavandula* “5 cm”	1	0.3935	–	–	–	0	–	23.009 (K$\Omega$)	551.16 (K$\Omega$)	–	–	5.9543 (nF)	199.55 (nF)	–	–	–
*Lavandula* “10 cm”	0.5271	1	–	–	–	0	–	585.57 (K$\Omega$)	9.7282 (K$\Omega$)	–	–	24.989 (nF)	0.4826 (nF)	–	–	–
Double-shell																
Marjoram “5 cm”	0.5649	0.9855	–	–	–	–	–	284.85 (K$\Omega$)	239.4 (K$\Omega$)	0	–	15.354 (nF)	0.9762 (nF)	–	–	–
Marjoram “10 cm”	0.7813	0.7044	–	–	–	–	–	1.6228 (M$\Omega$)	1.8514 (M$\Omega$)	94.061 (K$\Omega$)	–	7.9913 (nF)	1.5936 (nF)	–	–	–
Salvia “5 cm”	0.7524	0.6209	–	–	–	–	–	456.63 (K$\Omega$)	50.052 (K$\Omega$)	369.52 ($\Omega$)	–	1.5692 (nF)	7.9199 (nF)	–	–	–
Salvia “10 cm”	0.8041	0.6305	–	–	–	–	–	966.94 (K$\Omega$)	120.13 (K$\Omega$)	7.4007 (K$\Omega$)	–	0.6215 (nF)	6.2827 (nF)	–	–	–
*Lavandula* “5 cm”	0.3885	1	–	–	–	–	–	666.78 (K$\Omega$)	35.951 (K$\Omega$)	0	–	214.87 (nF)	5.2109 (nF)	–	–	–
*Lavandula* “10 cm”	0.5316	1	–	–	–	–	–	590.17 (K$\Omega$)	10.33 (K$\Omega$)	0	–	23.165 (nF)	0.5557 (nF)	–	–	–
Proposed Stem model																
Marjoram “5 cm”	1	0.6132	1	0.5402	0.4903	-	33.796 (M$\Omega$)	1.379 (M$\Omega$)	0	9.2607 (M$\Omega$)	43.527 (M$\Omega$)	0.7156 (nF)	7.7111 (nF)	0	41.614 (μF)	57.801 (nF)
Marjoram “10 cm”	0.7154	0.2225	1	0.0475	0.9561	-	2.0143 (M$\Omega$)	0	543.7 (M$\Omega$)	10.95 (M$\Omega$)	0	1.2829 (nF)	8.6658 (μF)	0.146 (nF)	31.372 (μF)	1.2404 (fF)
Salvia “5 cm”	0.6902	0.5903	0.8177	0.2834	1	–	460.62 (K$\Omega$)	1.0669 (K$\Omega$)	9.7919 (M$\Omega$)	284.34 (K$\Omega$)	10.663 (M$\Omega$)	1.9.99 (nF)	1.1963 (fF)	0.2533 (nF)	68.262 (μF)	5.9601 (fF)
Salvia “10 cm”	0.6129	0.8867	1	0.9857	0.7149	–	8.0791 (M$\Omega$)	2.3004 (M$\Omega$)	2.1541 (M$\Omega$)	3.4699 (M$\Omega$)	59.315 (M$\Omega$)	4.4953 (nF)	182.8 (nF)	0.3679 (nF)	82.203 (μF)	4.6292 (μF)
*Lavandula* “5 cm”	0.4788	0.1343	1	0.9360	1	–	48.259 (M$\Omega$)	0	84.606 (M$\Omega$)	536.39 (K$\Omega$)	39.186 (M$\Omega$)	59.305 (nF)	7.3663 (μF)	60.852 (fF)	8.863 (μF)	831.49 (nF)
*Lavandula* “10 cm”	1	0.4085	1	1	0.7986	–	66.724 (M$\Omega$)	2.0446 (M$\Omega$)	0	740.7 (K$\Omega$)	41.536 (M$\Omega$)	17.092 (fF)	63.123 (nF)	5.4559 (nF)	0	0.2967 (nF)
Proposed Simplified Stem model																
Marjoram “5 cm”	1	1	0.6737	–	–	–	7.0902 (K$\Omega$)	252.81 (K$\Omega$)	930.25 (K$\Omega$)	-	-	6.1565 (μF)	2.5936 (nF)	3.9351 (nF)	–	–
Marjoram “10 cm”	0.7396	1	0.7146	–	–	–	97.745 ($\Omega$)	938.9 (K$\Omega$)	24.342 (M$\Omega$)	-	-	39.234 (nF)	2.5913 (fF)	1.303 (nF)	–	–
Salvia “5 cm”	0.0883	1	0.6405	–	–	–	4.1162 (K$\Omega$)	134.14 (K$\Omega$)	2.8018 (M$\Omega$)	-	-	1.9577 (μF)	0.3668 (nF)	4.0499 (nF)	–	–
Salvia “10 cm”	0.714	1	1	–	–	–	35.391 (K$\Omega$)	162.47 (K$\Omega$)	953.3 (K$\Omega$)	-	-	1.1235 (nF)	35.002 (nF)	34.057 (fF)	–	–
*Lavandula* “5 cm”	1	1	0.4486	–	–	–	8.2413 (K$\Omega$)	434.87 (K$\Omega$)	641.19 (K$\Omega$)	-	-	8.3765 (nF)	54.624 (fF)	92.939 (nF)	–	–
*Lavandula* “10 cm”	1	0.1297	0.5844	–	–	–	596.96 ($\Omega$)	1.9615 (M$\Omega$)	532.04 (K$\Omega$)	-	-	22.461 (fF)	7.4162 (μF)	7.9638 (nF)	–	–

**Table 3:**
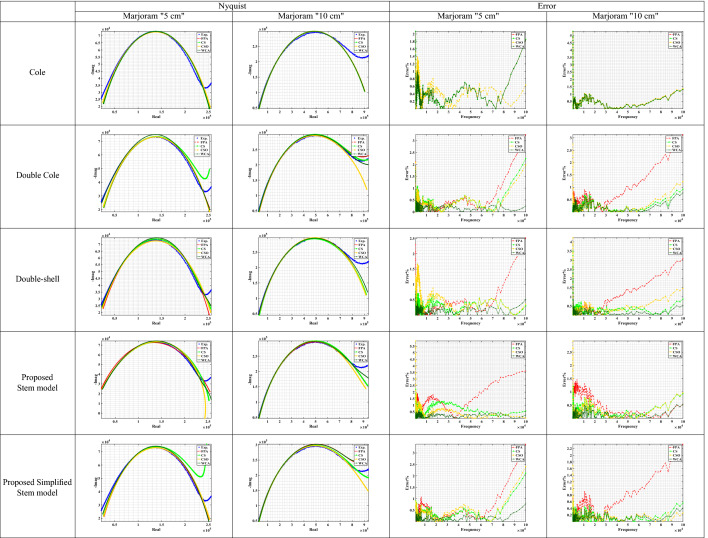
Marjoram Nyquist and Error plots of the experimental and the fitted models using different optimization algorithms.

**Table 4:**
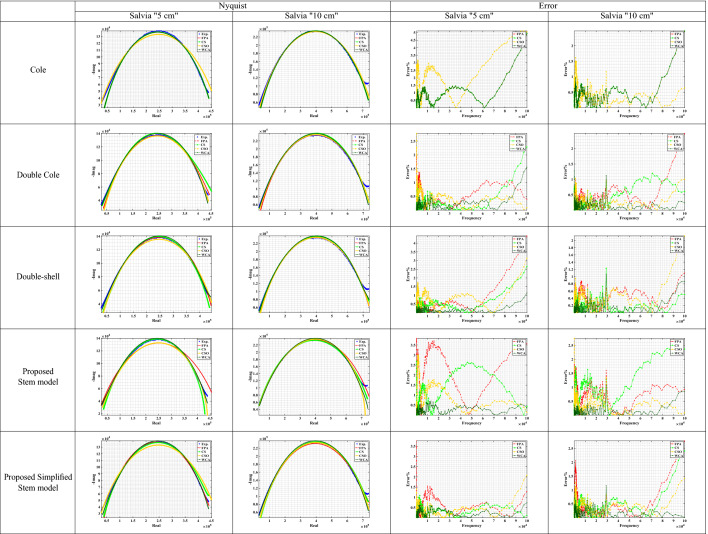
Salvia Nyquist and Error plots of the experimental and the fitted models using different optimization algorithms.

**Table 5:**
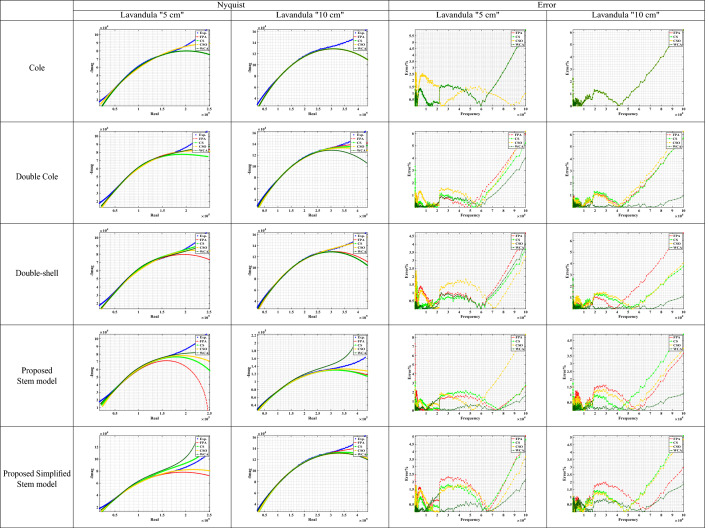
*Lavandula* Nyquist and Error plots of the experimental and the fitted models using different optimization algorithms.

For Marjoram plant samples, the Nyquist plot is plotted for the experimental data and the extracted parameters from the four applied optimization techniques (FPA, CS, CSO, and WCA), as shown in Table 3. The proposed stem model and the simplified model have shown good fitting results. Furthermore, the error between the measured impedance of marjoram by (SP150) and the fitted data is calculated for each model, where the FPA, CS, CSO, and WCA algorithms are applied for each model. WCA algorithm showed the best result compared to FPA, CS, and CSO optimizations in all studied cases. For the Marjoram plant samples at 5 cm distance, the maximum error calculated for Cole-impedance model is around 2%, for Double Cole is 2%, for Double-shell is 0.8%, for the proposed stem model is less than 0.5%, and for the proposed simplified stem model is less than 0.8%. For the case of Marjoram with a distance of 10 cm, the Cole-impedance model has a maximum error greater than 5%, for the Double Cole model is 0.8%, for the Double-shell is greater than 2.5%, while for the proposed model is 0.5%, and for the simplified one is less than 0.4%.

For Salvia plant samples, the Nyquist plot is plotted for the experimental data, and the extracted parameters from the four applied optimization techniques, as shown in Table 4. The proposed stem model and the simplified model have shown good fitting results. Furthermore, the error between the measured impedance of marjoram by (SP150) and the fitted data is calculated for each model, where the four optimization algorithms are applied for each model. WCA algorithm showed the best result compared to FPA, CS, and CSO optimizations in all studied cases. For samples at a 5 cm distance, the maximum error for the Cole model is around 5%, for the Double Cole model is around 1.6%, for the Double-shell model is 1.2%, while for the proposed stem model and the simplified one are less than 0.7%. While for the 10 cm distance, the maximum error for the Cole model is less than 2.5%, for the Double Cole model is equal to 1.3%, for the Double-shell model equals 1.1%, for the proposed model is around 1%, and for the proposed simplified model is around 1.1%.

For *Lavandula* plant samples, the Nyquist plot is plotted for the experimental data, and the extracted parameters from the four applied optimization techniques as shown in Table 5. The proposed stem model and the simplified model have shown good fitting results. Furthermore, the error between the measured impedance of marjoram by (SP150) and the fitted data is calculated for each model, where the four optimization algorithms are applied for each model. WCA algorithm showed the best result compared to FPA, CS, and CSO optimizations in all studied cases. for samples at 5 cm distance, the maximum error for Cole model is greater than 5.5%, for Double Cole and Double-shell models is 4%, for the proposed stem model is around 1.5%, and for the simplified stem model is around 2%. While for the 10 cm distance, the maximum error for the Cole model is greater than 6%, for the Double Cole model and Double-shell model is 2.5%, for the proposed stem model is around 1%, and for the proposed simplified stem model is around 1.8%.

For more exploration for the performance of the four used optimization algorithms, Convergence curves are investigated at 1800 iteration. Table 6 shows the convergence curves for a sample of Marjoram at 5 cm distance. In all models, WCA optimization converges at 1000 iteration, while CSO optimization shows an inconsistency behaviour. For CS optimization, it converges at 600 iteration for Cole and Double-shell impedance models, and converges at 1500 iteration for the Double Cole and the proposed stem models, while it needs more than 1800 iteration to converge in the proposed simplified stem model. For FPA optimization, Cole impedance model needs 1000 iteration to converge, and around 1700 iteration for Double-shell and the proposed stem model. While Double Cole impedance model converges at 1800 iteration, and the proposed simplified stem model needs more than 1800 iteration to converge. According to this results, WCA optimization outperforms the other three optimizations as it got the lowest error percentage in all cases. FPA and CS optimizations show defects when dealing with a bigger problem size; also, CSO showed an inconsistency behaviour in some cases.

The final outcome is that the proposed stem and the simplified stem models showed a remarkable performance over the commonly used models in plant stem tissues representations. Furthermore, WCA is the recommended technique for bio-impedance problems, especially for the larger problem size.

**Table 6:**
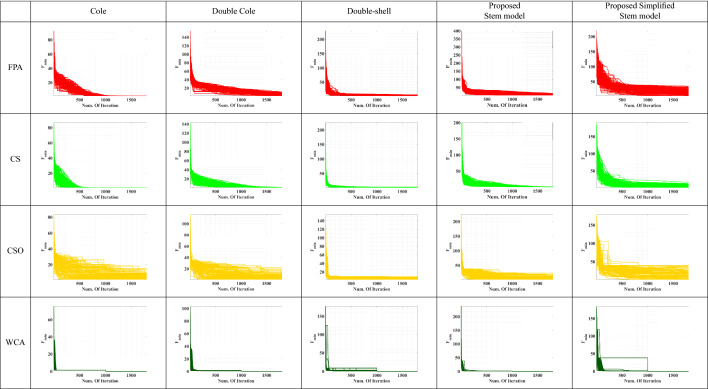
Convergence curve for all used optimizations for Marjoram sample.

## Conclusion

Two fractional-order bio-impedance models for plant stem characterization were introduced and compared with three known models (Cole, Double Cole, Fractional-Order Double-shell). Their parameters were extracted based on the measured data of three medical plant species using two samples each. The employed optimization algorithms are WCA and CSO optimizations, which are used for the first time in such problem, and compared with two conventional metaheuristic optimization techniques (FPA and CS) used before in similar problems. Error percentage was plotted versus frequency for each model using the four algorithms to test the models’ efficiency and the most efficient algorithm for the studied problem. The proposed models showed their significant advantage over the other used models in all electrode positions tested; they give the least fitting error percentage compared with the actual measurements’ data. The WCA optimization algorithm demonstrates its accuracy, particularly for a larger problem size where the FPA optimization algorithm shows some defects. Other metaheuristic optimization techniques were tested, such as Moth Flame Optimization (MFO), Black Widow Optimization Algorithm (BWOA), Whale Optimization Algorithm(WOA), Slime Mould algorithm (SMA), etc.. However, they did not match the WCA. For possible future work, more recent optimization algorithms can be employed in similar problems such as Polar Bear Optimization Algorithm (PBO)^66^, Red Fox Optimization (RFO)^52^, and Elephant Herding Optimization (EHO)^67^.
